# Oligonucleotides Carrying Nucleoside Antimetabolites as Potential Prodrugs

**DOI:** 10.2174/0929867328666211129124039

**Published:** 2022-01-24

**Authors:** Carme Fàbrega, Anna Clua, Ramon Eritja, Anna Aviñó

**Affiliations:** 1 Institute for Advanced Chemistry of Catalonia (IQAC), Spanish National Research Council (CSIC), Barcelona, Spain;; 2 Networking Center on Bioengineering, Biomaterials and Nanomedicine (CIBER-BBN), E-08034 Barcelona, Spain

**Keywords:** Antimetabolites, nucleosides, nanomedicine, antiproliferative oligomers, homopolymers, floxuridine, gemcitabine, cytarabine

## Abstract

**Background:**

Nucleoside and nucleobase antimetabolites are an important class of chemotherapeutic agents for the treatment of cancer as well as other diseases.

**Introduction:**

In order to avoid undesirable side effects, several prodrug strategies have been developed. In the present review, we describe a relatively unknown strategy that consists of using oligonucleotides modified with nucleoside antimetabolites as prodrugs.

**Methods:**

The active nucleotides are generated by enzymatic degradation once incorporated into cells. This strategy has attracted large interest and is widely utilized at present due to the continuous developments made in therapeutic oligonucleotides and the recent advances in nanomaterials and nanomedicine.

**Results:**

A large research effort was made mainly in the improvement of the antiproliferative properties of nucleoside homopolymers, but recently, chemically modified aptamers, antisense oligonucleotides and/or siRNA carrying antiproliferative nucleotides have demonstrated a great potential due to the synergetic effect of both therapeutic entities. In addition, DNA nanostructures with interesting properties have been built to combine antimetabolites and enhancers of cellular uptake in the same scaffold. Finally, protein nanoparticles functionalized with receptor-binders and antiproliferative oligomers represent a new avenue for a more effective treatment in cancer therapy.

**Conclusion:**

It is expected that oligonucleotides carrying nucleoside antimetabolites will be considered as potential drugs in the near future for biomedical applications.

## 1 INTRODUCTION

Cancer is a major healthcare problem as it is one of the leading causes of death worldwide. For this reason, the development of drugs for the treatment of complex diseases is one of the most important medical challenges for patients to benefit from increased treatment efficacy. Most of the therapies are non-selective, leading to side effects responsible for prolonged and costly recovery and often followed by relapse [1]. In this context, selective targeting of cancer cells should provide a platform for the development of less harmful treatments.

**Fig. (1):**
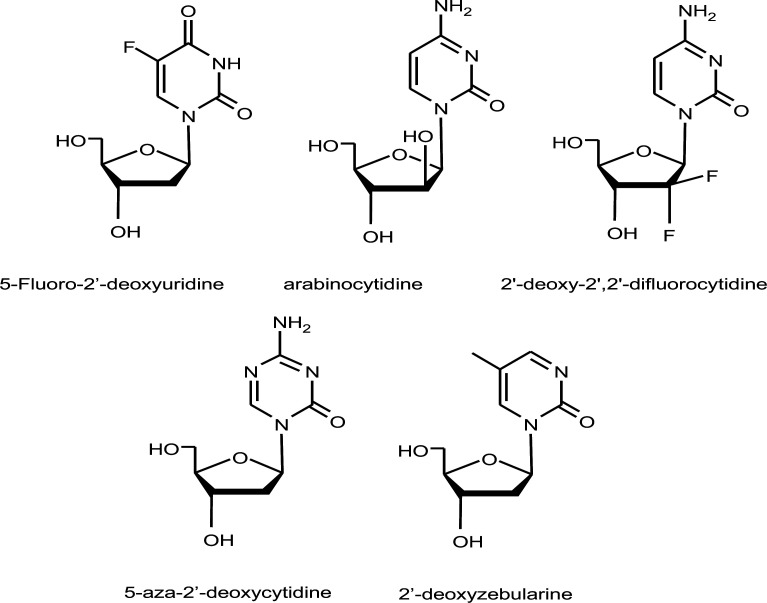
Nucleosides antimetabolites used in the treatment of cancer.

Therapeutic nucleosides and nucleobases are among the most widely used chemotherapeutic drugs against cancer, despite being one of the first compounds developed for the treatment of cancer malignancies. These include fluoropyrimidines, such as 5-Fluorouracil (FU) [2, 3] and its nucleoside derivatives (2’-deoxy-5-fluorouridine o floxuridine (FdU) and 5-fluorouridine); arabinonucleosides such as arabinocytidine (cytarabine, araC) [4], gemcitabine or 2’,2’-difluoro-2’-deoxycytidine [5] (Fig. **1**) and several purines, such as 6-thiopurines [6] and fludarabine [7]. Their antiproliferative mechanism of action involves the formation of the nucleoside-5’-monophosphate as the main active compound. The nucleoside monophosphate can be converted into the corresponding nucleoside-5’-triphosphate followed by its misincorporation into DNA or RNA by the DNA or RNA polymerases, respectively [8]. On the one hand, the modified DNA or RNA produces mutations yielding non-functional genomes [9]. On the other hand, nucleoside-5’- monophosphate can directly interact with the enzymes involved in nucleotide metabolism, causing the modification of the nucleotide pool. This, in turn, will increase the amount of mutated DNA and RNA. For example, floxuridine monophosphate reacts with the active center of thymidylate synthase, resulting in an irreversible covalent bond [10, 11]. This suicidal covalent reaction inhibits this enzyme, decreasing the thymidine phosphate in the nucleotide pool. This reduction induces thymine-less cellular death [12, 13]. In addition, FdU, araC, and gemcitabine modify DNA and can inactivate DNA topoisomerases [14].

Other modified nucleosides, such as 5-fluoro-2’-deoxycytidine [15], 5-aza-2’-deoxycytidine, decitabine [16], or 2’-deoxyzebularine [17], are able to inhibit cytidine-DNA methyltransferases (DNMT), an important group of enzymes involved in epigenetic regulation. The inhibition of DNMT has attracted considerable interest. The inhibition of aberrant DNA methylation patterns observed in cancer progression may activate tumor necrosis factors that help cells fight against tumors [18].

In addition to the therapeutic effects of these nucleosides, there are some undesirable side effects. One of the major problems described is cell resistance to the nucleoside antimetabolites in long treatments. Cancer cells become immune to these drugs mainly by increasing the rate of the corresponding nucleoside triphosphate biosynthesis [19], catabolism of the antimetabolite [20], and the antimetabolite efflux out of the cells [21]. The lack of specificity against cancer cells also leads to undesired side effects, such as toxicity towards the gastrointestinal tract, anemia, and dermatological and cardiac dysfunctions [2, 22]. In addition, different individual gene expression profiles [23] have been described. These drawbacks are mainly due to the large number of drugs needed to reach tumor target cells.

One potential solution is the use of nucleoside-containing lipid or peptide derivatives that act as prodrugs. Therefore, they are not active until they reach the desired target tissue, where they will be activated metabolically, avoiding the presence of drugs and their toxicity in healthy tissues. Some examples of this strategy are lipid-modified nucleoside and amino esters nucleoside prodrugs [24-26].

**Fig. (2):**
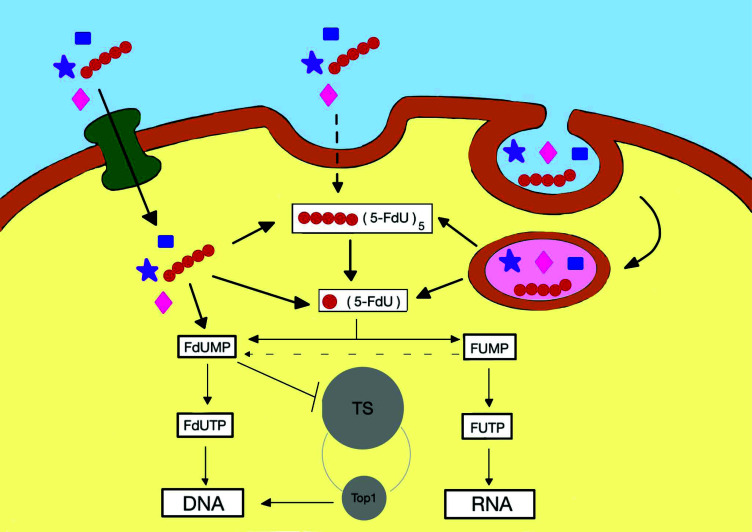
Schematic representation of the potential mechanism of action of oligonucleotides carrying antiproliferative nucleotides taking a FdU homopolymer as an example.

In the same way, another strategy that has been proposed is the use of oligomers or oligonucleotides carrying these antiproliferative nucleosides acting as prodrugs (Fig. **2**). The degradation by nucleases yields a mixture of nucleoside and nucleoside monophosphates as active compounds. In this case, the oligonucleotides are considered prodrugs since they are metabolically activated inside the cells avoiding systemic toxicity.

This strategy has been widely applied and recently improved with the incorporation of active and passive targeting units using nanoparticles, aptamers, DNA nanostructures, and nanoproteins. These new entities are achieving excellent results in terms of selectivity and efficacy, thereby avoiding potential toxicity. The oligonucleotide prodrug approach is particularly interesting as it combines the recent developments in therapeutic oligonucleotides with the new advances in nanomedicine and nanomaterials fields. The aim of this article is to review the application of oligonucleotides carrying nucleoside antimetabolites for the selective administration of these drugs for the treatment of malignancies from the first experiments performed at the end of the 20^th^ century to the most recent developments. To our knowledge, the development of nanosystems carrying nucleotide antimetabolites has not been previously reviewed.

## 2 OLIGONUCLEOTIDES CARRYING FLOXURIDINE (FdU)_10_

FdU_10_ is a prodrug for the intracellular release of 5-fluoro-2’-deoxyuridine monophosphate (FdUMP). It was designed to overcome the medical need for more effective fluoropyrimidine (FP) drugs. It has an enhanced anticancer activity, reduced systemic toxicities, and improved pharmacological outcome relative to current fluoropyrimidines and 5-fluorouracil (FU) [27]. FdU_10_ is an oligonucleotide sequence of 10 molecules of FdU used to avoid the intracellular conversion of FU to its active metabolite [3]. The cytotoxic effect of FdU_10_ is a consequence of the multimeric form uptake and stability, and it is independent of OPRTase or TK expression [28].

FdU_10_ exhibits a more effective antiproliferative action than FdU monomer, and it is notably more active than FU in a comparative analysis performed in 60 cancer cell lines from the NCI [29]. In addition, FdU_10_ cytotoxic activity was correlated with positive expression of several genes involved in endocytosis, such as clathrin, SNF8, a component of the endosomal sorting complex, or annexin A6 implicated in membrane related events, indicating a protein-mediated cellular uptake of FdU_10_ prodrug [30]. This active transport facilitates the high concentration of FdU monomer inside the tumoral cells, causing massive DNA damage and apoptosis by the inhibition of thymidylate synthase (TS) and topoisomerase 1 (Top1) poison [30, 31]. The inhibition of TS provokes the reduction of thymidine (Thy) levels and the accumulation of dUMP, the TS substrate, producing an imbalance in the nucleotide triphosphate (dNTPs) pool. As a consequence, the dUTP levels increase, facilitating its incorporation into DNA [32]. In parallel, the presence of FdUTP induces the incorporation of FdU nucleotide to DNA, causing irreparable DNA damage [27]. Top1 efficiently cleaves the DNA-misincorporated FdU; however, FdU prevents the religation step of Top1 catalysis from generating the formation of Top1 cleavage complex (Top1cc) [29, 33]. This complex is also formed and is then responsible for the poisoning of other nucleosides analogs, such as araC [14, 34] and gemcitabine [14, 35]. The dual mechanism of action targeting TS and Top1 makes FdU_10_ a more potent therapeutic drug than 5-FU against different human cancer types [27, 29]. TS inhibition is produced at the lowest concentration needed for FdU_10_ cytotoxicity [36, 37], avoiding a feedback effect of new TS being synthesized [38] as TS is an RNA-binding protein that is regulated by multiple genes, including its own expression [39]. This inhibition has been demonstrated to persist for sufficient time, facilitating cell death [27, 37]. Rescue of the thymineless conditions produced by FdU_10_ with exogenous thymidine is only efficient within the first 18 hr of treatment previously to cell replication and division, where the misincorporation of dU and FdU into DNA causes irreparable damage and the formation of Top1cc is not prevented [40].

The differences in the mechanism of action of FdU_10_ versus previous FPs make it have an exceptional activity against a spectrum of tumoral cells, including leukemia [31, 40-42], glioblastoma [43], prostate cancer [37], and the central nervous system (CNS) malignancies [29, 30]. FdU_10_ has demonstrated antileukemia activity as a single agent compared to the treatment with doxorubicin and araC [31], presenting a clearly reduced systemic toxicity compared to the combination of these two drugs [31]. Similar results were obtained against preclinical acute lymphoblastic leukemia (ALL) models [41]. FdU_10_ was rapidly internalized, with low toxicity, exhibiting high efficacy against all the ALL cells, including the cells resistant to araC [41]. Previous studies reported that FdU_10_ in acute myelogenous leukemia (AML) induces thymineless death *via* activating the extrinsic apoptotic pathway and reducing overall levels of lipid rafts [44]. These results suggested a role of lipid rafts in cell death by co-localization of Fas and Fas ligand (FasL) in the plasma membrane [44]. In preclinical models of prostate cancer, the treatment with FdU_10_ not only increased survival by significantly decreasing tumoral growth but also made them sensitive to radiation. Furthermore, it was found to be well tolerated with minimal systemic toxicity [37]. The pro-apoptotic effect of FdU_10_ towards prostate cancer can be enhanced by the modulation of the intracellular levels of Zn^2+^, as this ion is involved in the survival/apoptosis balance mechanism [45]. Due to the cytotoxicity displayed by FdU_10_ to the CNS malignancies [31] and its ability to cross the blood-brain barrier, its effect was also evaluated in human Glioblastoma (GBM) through local administration. The efficacy of FdU_10_ in eradicating the GBM xenograft model without damaging normal brain tissue was found to be strong, including the proximal to the tumor mass [43]. In contrast, traditional fluoropyrimidines such as 5-FU produced serious neurotoxicity at equivalent doses [46]. Due to the local administration, the total dose necessary was considerably low compared to the dose used in the systemic treatment of leukemia [31]. The cytotoxic effect in the glia cells was produced by TS inhibition and induction of Top1cc formation, which resulted in DNA damage and cell death. Thymidine rescue experiments reversed FdU_10_ cytotoxicity and Top1cc formation only during the first 18h of treatment prior to DNA replication [43]. A comparative study on colorectal cancer (CRC) comparing FU and FdU_10_ reveals that the oligomer is more potent than FU in CRC, independently of the TP53 status [47]. In addition, FdU_10_ decreases replication fork velocity and causes replication fork collapse in CRC cells, whereas 1000-folds excess of FU is necessary to accomplish analogous endpoints. This effect can be enhanced by combining FdU_10_ with Chk1 inhibitors [42]. Several studies proved that FdU_10_ produces minimal systemic toxicities, including gastrointestinal (GI) toxicity, compared to FU [48]. All these results are consistent with a nearly exclusive DNA-directed mechanism [27].

Combining the FdU oligomer with other therapeutic compounds could enhance its cytotoxic effect. Considering this principle, a different number of FdU monomers were incorporated to the 3’ end of a telomerase inhibitor antisense oligonucleotide (ASO) and tested in human fibrosarcoma and colon adenocarcinoma cell lines. The (FdU)_n_ conjugated presented an antiproliferative activity higher than FdU in both cell lines [49]. TS inhibition is a part of the mechanism of action of FdU_10_ due to the release of the FdU monomer. For this reason, the dual strategy based on the combination of small interfering RNAs (siRNAs) that target TS and the presence of FdU moieties will lead to an enhancement of the cytotoxicity by inducing DNA damage and the apoptotic process [50]. However, the position of the FdU has to be selected carefully as its incorporation within the antisense strand interferes with the efficiency of the siRNA. Moreover, to increase the cell stability of FdU_10,_ a second-generation of the oligonucleotide containing FdU has been synthesized. The modification introduced to reduce exonucleolytic degradation consists of the incorporation of an arabinosyl cytidine (AraC) to the 5’-end with a polyethylene glycol linker connected to the FdU units. This new entity, CF10, enhances TS inhibition and Top1 cleavage complex formation [51]. Also, CF10 was found to be more effective than 5-FU and cisplatin in a patient-derived organoids study of SCLC [14]. Similar results have been obtained in multiple colorectal cancer cell lines, being even more effective than the prototype FdU_10_ polymer [52]. CF10 is in an early-phase clinical trial for the treatment of SCLC and colorectal cancer (Table **1**).

## 3 OLIGONUCLEOTIDES CONTAINING ANTI- PROLIFERATIVE NUCLEOSIDES CONJUGATED WITH LIPIDS, CARBOHYDRATES, AND APTAMERS

**Fig. (3):**
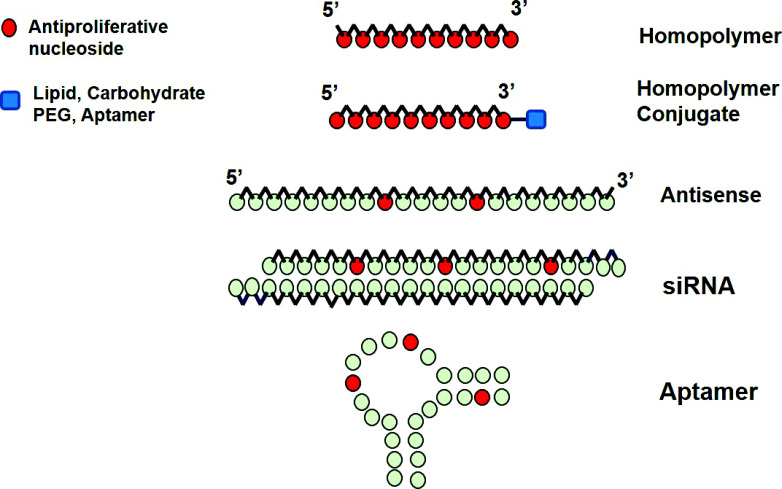
Schematic representation of diverse oligonucleotides that have been functionalized with antiproliferative nucleosides.

Solid-phase synthesis of nucleic acids is based on the building blocks of nucleoside phosphoramidites to construct oligonucleotides with a well-defined and tunable molecular structure. This powerful technology has been applied to synthesize therapeutic oligonucleotides based on antisense or siRNA approaches. Nucleic acids analogues carrying antimetabolites in their sequences have also emerged as powerful drug modules for engineered oligonucleotides (ODN) with therapeutic applications. FdU and araC are commercially available as phosphoramidite monomers, so they can be included in oligonucleotide synthesis cycles to prepare drug-oligonucleotide hybrid molecules or homopolymer antitumor drugs as presented in the previous section (Fig. **3**).

FdU has been incorporated in antisense oligonucleotides and then conjugated to paclitaxel to form micelles and nanoparticles. These conjugates directly knock down the expression of P-glypoprotein and release FdU and paclitaxel with a synergistic effect to inhibit tumor growth [53].

Another example of FdU incorporated in therapeutic ODN was the thymidylate synthase-targeted siRNAs that contained 5-fluoro-2′-deoxyuridine (FdU) moieties at various locations within the siRNA (Fig. **3**). The designed siRNA has enhanced siRNA cytotoxicity once FdU is released [50].

Similarly, gemcitabine has been introduced to replace cytidines in a series of siRNAs designed to improve biological activity in gene silencing experiments. The modifications, located in six different positions of the antisense strand, were crucial for siRNA silencing activity, indicating a position-dependent tolerance for this modification. The introduction of this analogue had little impact on the structure of the siRNA duplexes [54, 55]. More recently, several units of gemcitabine have been introduced to siRNAs in the sense strand, demonstrating the synergism between the two drugs. This results in a single drug molecule that simultaneously co-delivers gemcitabine and the synergistic siRNA [54]. The authors demonstrated a 5-30-fold improvement in potency compared to gemcitabine alone in a wide array of pancreatic tumor cells and may represent a novel therapeutic approach for treating pancreatic cancer.

On the other hand, the group of Gmeiner proposed a DNA hairpin containing FdU units that could interact through the minor groove binder with curcumin (Fig. **3**). The stable complex showed increased cytotoxicity combined with FdU in cancer therapy [56].

Several efforts have been devoted to increasing the number of systems to deliver FdU-containing oligonucleotides. For this reason, conjugation of different active molecules has been reported in several studies (Fig. **3**). In this direction, the conjugation of lipids to the FdU_20_ oligomer has been described [57]. The aim of these conjugates is to improve the delivery of the oligomer by adding two octadecyl chains inserted into the core of albumin by hydrophobic interactions. The complex lipid-FdU_20_/albumin accumulates in the tumor through enhanced permeability and retention (EPR) and is internalized into lysosomes. After enzymatic degradation, floxuridine monophosphate is released to inhibit cell proliferation [57].

Recently, our group has described the conjugation of small molecules as potential delivery enhancers, such as lipids (cholesterol, palmitic acid), receptor ligands (folic acid, N-acetylgalactosamine, GalNAc), and polyethylene glycol (PEG), to (FdU)_5_ oligomer for improving their pharmacological properties [58]. The conjugates were straightforwardly synthesized over the corresponding enhancer molecules attached to solid supports and the FdU phosphoramidite. Most of the delivery enhancers were placed at the 3’-end of the oligomers except for folic acid that was added at the 5’-end. The cytotoxicity and apoptosis assays showed that the most active were the best-internalized oligomers. In this case, the best conjugates were those carrying palmitic or folic acid ligands with increased cytotoxicity in FdU-resistant colorectal cancer cells. These modifications also stabilized the degradation of FdU_5_ in the bloodstream without preventing the metabolic activation of these polymeric drugs inside tumoral cells [58].

The carbohydrate GalNAc ligand recognizes the specific surface asialoglycoprotein receptor (ASGR) that is over-expressed on the cell surface of hepatocytes. Antisense ODN and siRNA modified with GalNAc are in advanced clinical trials. Conjugation of this ligand to oligonucleotides facilitates their productive uptake. Palmitic acid conjugation has been considered an excellent alternative for extrahepatic delivery of therapeutic oligonucleotides [59] with enhanced internalization and increased endosomal release [60].

Among ligands for targeted therapy, nucleic acids aptamers have also been considered attractive molecules to improve delivery. Aptamers are short single-stranded oligonucleotides with specific recognition abilities to bind to a given receptor molecule. Similar to antibodies, they have been used for diagnostic and therapeutic applications [61]. However, aptamers are usually smaller than antibodies, resulting in the good ability to penetrate tissues, and no immunogenic activation has been reported in most *in vivo* experiments. In addition, as they are chemically synthesized, several modifications can be introduced to improve the pharmacologic properties.

Aptamers may fold into various secondary structures (for example, stem-loop duplexes, G-quadruplex), forming unique three-dimensional structures that are capable of specific recognition to their targets. Binding affinities are in the range of pM-nM, comparable to those of other molecular ligands. The targets range from small molecules and proteins to cells [62]. For therapeutic applications, aptamers have been developed against a broad spectrum of diseases, including cancer, diabetes, or aids [63].

The conjugation of chemotherapeutic drugs to tumor-targeting aptamers can increase the drug delivery with the purpose of reducing the exposure of non-target sites to chemotherapy agents [64]. Their chemical and thermal stability and conformational reversibility enable the production of different programmable designs of aptamer drug conjugates. The conjugates are internalized by means of endocytosis that are conveyed into lysosomes. The hydrolysis of these prodrugs by nucleases would yield the active drugs released by active nucleoside transporters within the targeted cells exclusively while sparing normal cells (Fig. **2**).

Two main approaches are reported following this purpose. The first approach involves attaching the drug formed by polymeric antimetabolite nucleosides in the 3’- or 5’-ends of the aptamer. The enzymatic degradation of the polymer generates an active single chemotherapeutic drug for specifically targeted cells. In pancreatic cancer, a nuclease-resistant RNA aptamer, SQ2, which binds to alkaline phosphate placental-like 2 (ALPPL2), a putative biomarker, is conjugated to a FdU-pentanucleotide FdU_5_ at the 3’-end connected through a phosphorothioate backbone. SQ2- FdU_5_ conjugate is internalized and can deliver FdU specifically to ALPPL2-expressing pancreatic cells, inhibiting cell proliferation [65].

Most pancreatic cancers overexpress epidermal growth factor receptor (EGFR), a transmembrane receptor tyrosine kinase. Nuclease resistance RNA aptamer, E07, in association with polymeric gemcitabine generated by polymerization, is delivered to EGFR-expressing cells, leading to the inhibition of cell proliferation [66].

The second approach consists of incorporating FdU and gemcitabine in the same sequence of the aptamer used for targeted delivery. For example, the synthesis of a gemcitabine-modified GPC3 aptamer is recently reported. This aptamer targets Glypican-3 described as a biomarker for hepatocellular carcinoma. Three consecutive gemcitabines were introduced in one of the loops of the aptamer. The results of the modified aptamer showed significant antitumor effects against tumors GPC3-positive, suggesting that this strategy represents a promising treatment for hepatocellular carcinoma [67]. The same approach was exploited to prepare the gemcitabine modified aptamer APTA-12. Based in the AS1411 sequence, this aptamer adopts an intramolecular G-quadruplex structure with single gemcitabine incorporated in the loop. The modified aptamer was designed to target nucleolin, an over-expressed membrane protein present in several cancer cells. The therapeutic activity of the modified aptamer was assessed in pancreatic cancer cell lines. In addition, this aptamer was also evaluated *in vivo* with good results for the growth inhibition in pancreatic Captan-1 tumor-bearing mice. The same aptamer was also used in combination with doxorubicin in breast cancer cells, showing synergetic and selective effects [68]. The synthesis of modified aptamer with multiple modified nucleosides was also obtained by an enzymatic reaction. For example, an AIR-3 aptamer with 30 units of FdU was prepared by transcription in the presence of FdUTP instead of UTP using a specific T7 RNA polymerase. This RNA aptamer bound the human interleukin-6 receptor that was internalized and then degraded and released [69]. The group of Rossi constructed enzymatically two pancreatic cancer-specific RNA aptamers that included gemcitabine and FdU triphosphates, respectively. This aptamer was found to deliver chemotherapeutic agents specifically to pancreatic ductal adenocarcinoma with reduced side effects [70].

## 4 HOMOMERIC OLIGONUCLEOTIDES CARRYING ANTIPROLIFERATIVE NUCLEOSIDES CONJUGATED TO PROTEINS

Protein nanoparticles have opened up a wide range of opportunities in the nanomedical field. Their tunable structural and functional versatility by genetic engineering has resulted in the synthesis of successful molecules for targeted therapy. The conjugation of polymeric antimetabolites to these particles has resulted in an excellent strategy for delivering the cargo molecules into the appropriate intracellular compartment of target cells. The group of Villaverde has exploited this approach by applying CXCR4-targeted self-assembled protein nanoparticles. These genetically engineered proteins have been chemically reacted with FdU oligomers, and the resulting protein conjugates were used for delivering FdU to target cancer cells. Specifically, protein nanoparticles based on the green fluorescence protein (GFP) incorporating the T22 peptide, a known ligand of the chemokine receptor CXCR4, are associated with metastatic progression in several tumor types such as colorectal cancer [71].

**Fig. (4):**
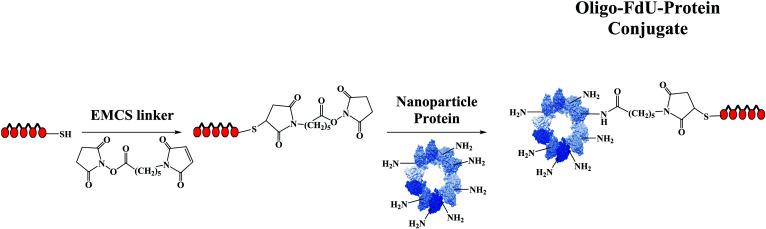
Chemical reactions used in the functionalization of nanoproteins with FdU-oligonucleotides.

The nanoconjugate was obtained by the reaction of the Lys of the protein and the thiol group introduced at the 3’-end of the pentameric-FdU using a bifunctional linker (Fig. **4**), [72]. The resulting therapeutic protein T22-GFP-H_6_-(FdU)_5_ has antimetastatic activity as it selectively delivers FdU by the degradation of the polymeric FdU attached to the protein in CXCR4^+^ cells. This targeted drug approach yields more effective and selective treatment than free (FdU)_5_. Our group, with the aim of producing more active conjugates and higher batch-to-batch reproducibility of the nanoconjugate, found that the best strategy for the preparation of these conjugates is to first perform the reaction of the bifunctional linker with the thiolated oligo-FdU_5_ and, then, the conjugation of the resulting maleimido-FdU_5_ with the protein through the amide formation [73].

In a step forward for searching more effective cytotoxic nanoparticles, dual-acting FdU_5_ and monomethyl auristatin E (MMAE) were chemically coupled with the same GFP scaffold protein against cancer cells [74].

The same authors explored the use of oligonucleotide FdU covalently attached to protein nanoparticles as an antimicrobial entity. Previously, it was found that the nucleobase 5-fluorouracil has relatively high antibacterial activity and could be used to prevent bacterial plaque formation on surfaces, such as catheters and other surgical instruments [75]. Therefore, the authors considered the possibility of linking the FdU oligomers to antibacterial peptides that can disrupt the bacterial membrane in an appropriate protein scaffold. To this end, two engineered proteins were able to form nanoparticles with two different antimicrobial peptides. Combining the peptide with the oligo-FdU on the protein scaffold produced an innovative nanomedicine drug, which can be considered a potential alternative to conventional antibiotics [76].

A potential drawback of using GFP-derived nanoproteins is the potential stimulation of the immune system as GFP is not a human protein. Recently, this problem has been addressed by replacing the GFP nanocarrier with a human nidogen-domain protein (HSNBT). The new conjugate, T22-HSNBT-H_6_-FdU_5,_ presents efficient drug transportation, thus providing an improved therapeutic window for CXCR4-targeted cells compared to T22-GFP-H_6_-FdU_5_ [77].

## 5 OLIGONUCLEOTIDES LINKED TO DNA NANOSTRUCTURES

**Fig. (5):**
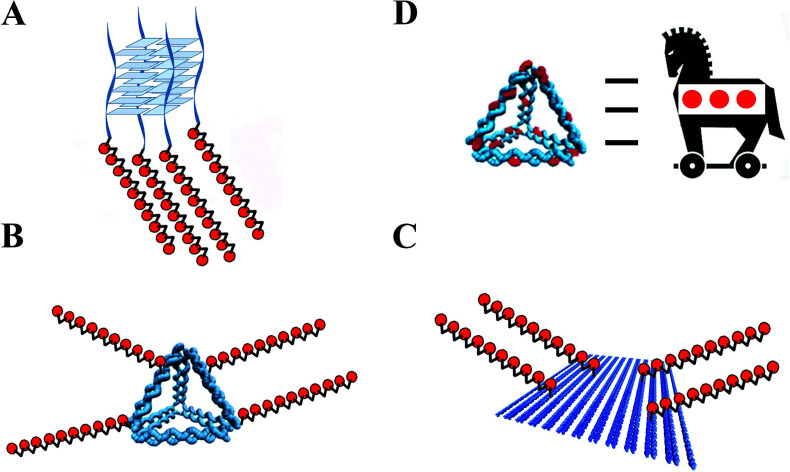
Schematic representation of DNA nanostructures functionalized with antiproliferative nucleotides.

DNA nanotechnology exploits the molecular recognition and capability of programmable self-assembly of the nucleic acid molecule to create well-defined nanoarchitectures [78, 79]. These DNA nanostructures offer great potential as drug delivery carriers [79, 80]. Floxuridine can be incorporated in DNA nanostructures at any position through its integration into the nucleic acid strand responsible for the formation of the DNA nanostructures (Fig. **5**). Recently, two parallel G-quadruplex TG_4_T or TG_6_T containing a sequence of (FdU)_5_ at the 5'-termini have shown an efficient internalization in tumoral cells. The addition of the G-quadruplex structures increased the cytotoxic effect of the FdU oligomer (Fig. **5A**) [81]. The role of the G-quadruplex nanostructures was to facilitate the cellular uptake of the FdU pentamer by interacting with protein receptors present in cancer cells helping to circumvent the cellular FU-resistant mechanisms. The incorporation of the 5 units of FdU at the 5'-end did not affect the formation of the G-quadruplex parallel structures.

An important issue in the efficacity of DNA drugs nanocarriers is the specific delivery to the tumoral cells. For this reason, incorporating an antibody-like molecule, affibodies as active transporter to a DNA nanoparticle, could increase the specificity for tumor cells. HER2 breast cancer cells were selectively inhibited by a DNA tetrahedron containing FdU polymeric tails. This construct also has affibody molecules for targeting HER2 attached to one of the FdU tails [82]. This strategy can expand to different cancer therapy by using other nucleosides analogs and other types of affibody molecules.

Two DNA nanoscaffolds (Figs. **5B** and **5C**), a DNA tetrahedron and a rectangle DNA origami, were functionalized with FdU_n_ oligomers and cholesterol moieties, exhibiting enhanced cytotoxicity and a higher capability to trigger apoptosis compared to the conventional 5-FU and FdU, particularly when cholesterol was used as internalization helper [83]. The FdU_n_ oligomers and the cholesterol units were synthetically attached to the end of the tetrahedron and DNA origami staples using solid-phase synthesis technology. These two DNA nanostructures carrying FdU_n_ were able to circumvent the 5-FU low sensitivity of colorectal cancer cells. DNA nanoscaffolds resulted as promising drug delivery nanocarriers for cancer treatment. On the other hand, Floxuridine is structurally similar to thymidine. This analogue can replace the thymidines in the DNA strands used for assembling the polyhedral DNA nanostructure without distorting this structure. This new strategy mimics the Trojan Horse for the protection and delivery of anticancer drugs (Fig. **5D**). These drug-container nanostructures exhibit superior anticancer ability compared to the free drug both *in vitro* and *in vivo* [84].

## 6 OLIGONUCLEOTIDES CARRYING DNA METHYLTRANSFERASE INHIBITORS

**Fig. (6):**
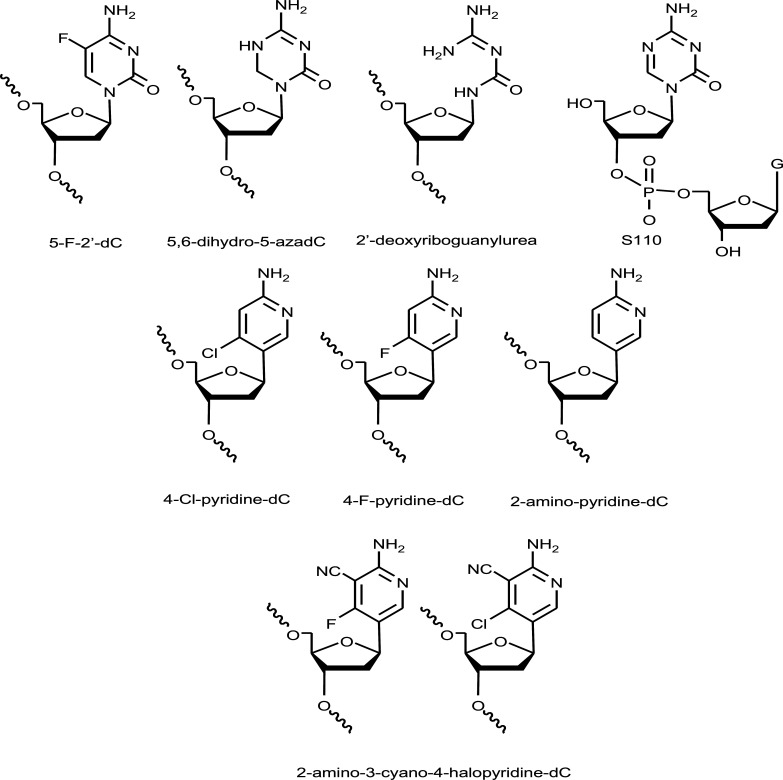
Chemical representation of some DNA methyl-transferase inhibitors, including the dinucleotide S110.

DNA cytosine 5-methyltransferases (DNMT) are a group of enzymes that catalyze the transfer of a methyl group from S-adenosyl methionine (SAM) to the carbon-5 position of cytosine residues in CpG sequences. This reaction is especially relevant in gene regulation as the methylation of CpG sequences is an epigenetic signal that triggers gene inhibition. DNA methylation patterns change in many cancer cells as a result of aberrant DNA methylation provoked by alterations in the concentration of DNMT isoforms, and some genes involved in tumor suppression could be hypermethylated [18]. For several reasons, the quest for DNMT inhibitors has been an important matter of research, especially in the development of selective inhibitors towards particular DNMT isoforms involved in cancer progression. The first nucleoside derivatives incorporated into DNA that were found to be potent DNMT inhibitors were 5-fluoro-2’-deoxycytidine (Fig. **6**), [15] and 5-aza-2’-deoxycytidine or decitabine (Fig. **1**), [16]. DNA modified with these nucleosides reacted covalently with DNMT in living cells, inhibiting tumoral growth [85]. 5,6-Dihydro-5-azacytosine (Fig. **6** [86]), zebularine (Fig. **1**) [17, 87], 2’-deoxyriboguanylurea (Fig. **6**), [88] and some conformationally restricted abasic sites [89] were found to act as mammalian DNMT inhibitors. Moreover, a series of 2-amino-4-halopyridine-C-nucleosides with electron-withdrawing groups (Fig. **6**) have been developed as nucleotides that are susceptible to stimulating nucleophilic aromatic substitution and increasing the covalent reaction between the cysteine of the active center of DNMT and the modified oligonucleotide inhibitor [90, 91].

The most widely used DNMT inhibitors used in clinical practice are 5-azacytidine and 5-aza-2’-deoxycytidine. These compounds were initially found in the anticancer drugs, but later it was demonstrated that the mechanism of action of these compounds was the inhibition of DNA methylation. These compounds have several drawbacks, including instability in aqueous solutions, high toxicity, and metabolic deamination to inactive 5-azauridine derivatives. The delivery of 5-aza-2’-deoxycytidine to cells was improved by using dinucleotides, trinucleotides, and tetranucleotides carrying one or two 5-aza-2’-deoxycytidine, thereby increasing the stability of the drug in water, lowering the toxicity, and protecting this nucleotide from cytidine deaminase [92]. It was demonstrated that these short oligonucleotides could generate the 5’-monophosphate 5-aza-2’-deoxycytidine derivative by nuclease degradation or directly inhibit DNMT by forming covalent bonds with the active site of the protein. From this study, the dinucleotide Guadecitabine (S-110 or SGI-110) (Fig. **6**) consisting of 5-aza-2’-deoxycytidine and 2’-deoxyguanosine linked by a 5’-3’phosphate bond was selected for its evaluation in phase III clinical trials (Table **1**) [93, 94].

## 7 PRECLINICAL AND CLINICAL TRIALS OF OLIGONUCLEOTIDES CONTAINING NUCLEOSIDE ANTIMETABOLITES

The biodistribution, biosafety, and antitumoral activity of some of the drugs described in this article have been evaluated through mouse cancer models in different preclinical trials. The results of these studies are summarized in Table **1** [97]. Moreover, two of them, CF10, the second generation of fluoropyrimidine polymer, and Guadecitabine (SGI-110), formed by 5-aza-2’-deoxycytidine and 2’-deoxyguanosine, have entered in clinical trials. The excellent *in vivo* data showed by these compounds in clinical trials may be envisaged as a future opportunity for other oligonucleotide containing nucleoside antimetabolites in cancer therapy.

## CONCLUSION

Nucleosides and nucleotides are important sources of compounds with important therapeutic applications as antiproliferative, antiviral and/ or antibacterial properties [95, 96]. The extensive research done by the group of Gmeiner and others on the antiproliferative activity of FdU_10_ oligomer has stimulated recent research on the use of homopolymers and oligonucleotides carrying antiproliferative nucleosides. These studies have included the development of synergetic therapeutic oligonucleotides, DNA nanostructures, and DNA-protein nanoconstructs with exquisite active and passive delivery systems as well as *in vivo* targeting functions. The excellent results obtained so far will probably encourage a deeper exploration of these prodrug strategies in order to generate exciting nanomedicines for personalized medicine with lower toxicity and precise targeting. Considering that only one dinucleotide has been tested in advanced clinical trials, we believe that the next challenge in this field will be to provide defined nanoconstructs containing antiproliferative nucleotides with optimized and reproducible protocols.

## Figures and Tables

**Table 1:** Oligonucleotides carrying antiproliferative nucleosides in preclinical and clinical trials.

**Preclinical Trials**										
**Drug**		**Model and Condition**		***In Vivo* Studies**			**Effects**		**Refs.**
FdU_10_		Balb/c nude mice leukemia model		toxicity			Less toxicity than 5-FU		[28]
FdU_10_		Mice leukemia model		Activity and toxicity			Higher survival, Less toxicity than 5-FU or Ara/Dox		[31]
FdU_10_		Mice xenograft PC3, prostate cancer		Activity			Higher survival, Enhancement of radiotherapy		[37]
FdU_10_		Mice leukemia model		Activity			Increased survival		[41]
FdU_10_		Mice xenograft Glioblastoma		Activity			Tumor regression Minimal toxicity		[43]
PTX-ASO-FdU (Chemogene)		Balb/c nude mice Drug resistant HeLa tumors		Biodistribution, activity and toxicity			Tumoral inhibition		[53]
lipid-FdU_20_/albumin		Balb/c nude mice tumor-implanted		Biodistribution, activity, and toxicity			Tumor growth inhibition		[57]
G12msi		Balb/c nude mice of hepatocellular carcinoma		Biodistribution, activity, and toxicity			Tumor growth inhibition		[67]
T22-GFP-H_6_-FdU		Colorectal cancer mouse model		Biodistribution, activity, and toxicity			Tumor growth inhibition Targeted delivery, antimetastatic effect		[72]
Buckyballs-FdU		Mice with hela cells injected		Biodistribution and activity			Tumor accumulation Anticancer activity		[84]
Affibody-FdU_n_		Mice ductal carcinoma		Biodistribution and activity			Tumor accumulation Antitumoral efficacity		[97]
**Clinical Trials**									
**Drug**	**Condition**		**Target**		**Sponsor**	**Status**		**Identifier**		
CF10	SCLC and colorectal cancer		TS		Wake	Prospective study		NCT03741829		
SGI-110	MDS or AML		DNMT		Astex	Phase 3		NCT02907359 NCT02920008		

FdU_10_: Polymer of 10 units of 5-fluoro-2’-deoxyuridine monophosphate; PTX-ASO-FdU/Chemogene: Paclitaxel-antisense oligonucleotide with FdU; G12msi: a gemcitabine-incorporated DNA aptamer, CF10: Second-generation fluoropyrimidine polymer; SCLC: Small Cell Lung Cancer; TS: Thymidylate synthase; MDS: Myelodysplastic Syndromes; AML: Acute Myelogenous Leukemia, DNMT: DNA methyltransferase; Wake: Wake Forest University Health Sciences; Astex: Astex Pharmaceuticals, Inc.
